# Tests for Albumins

**Published:** 1899-12

**Authors:** Byron H. Daggett

**Affiliations:** Buffalo, N. Y.


### TESTS FOR ALBUMINS.

A LECTURE DELIVERED BEFORE THE SCHOOL OF NURSES, BUFFALO HOSPITAL SISTERS OF CHARITY, OCTOBER 25, 1899.

By BYRON H. DAGGETT, M. D., Buffalo, N. Y.

ALBUMIN is not a normal ingredient of the urine, hence its presence in this secretion indicates some disturbance of function, if not some organic lesion. Our purpose today is to study methods of testing for albumin in the urine and of the ways of differentiating its various forms, rather than to discuss its significance or its sources.

Albumin is a proximate principle, a product of life, an amorphous, crystalisable substance, formed of carbon, hydrogen, nitrogen, oxygen and sulphur. It is soluble in strong acids and alkalies. Albumin is a complex substance and is arranged by both animal and vegetable life.

It occurs in the egg, blood, lymph, chyle, all serous fluids, juices of the flesh, in the brain, the pancreas and in all transudations from the bloodvessels and lymphatics. Its elements are the same under all conditions, only varying in their relative proportions If it does not contain all of these elements it is not albumin. It mixes with other substances without disintegration. There are different forms of albumin, possessing varying properties, due to the different proportions of its elements, just as there are different forms of alcohol and other hydrocarbons, due to the varying properties of their essential elements, carbon, hydrogen and oxygen.

Serum albumin, occurring in albuminuria, is derived from the blood serum. It resembles egg albumin and chemically it is a weak acid. It is not coagulated by water and with difficulty by hydrochloric acid. It is readily precipitated by nitric acid and is redissolved by an excess of the reagent. Serum albumin is an essential part of the blood. Its persistent presence in the urine, or its existence in quantities more than a trace indicates serious disease; the greater the quantity the more grave is the condition, although in contracted kidney it is absent at times or present in small quantities.

The sifting of serum albumin through the kidneys in amounts exceeding 1-30 to 1-40 per cent. is a storm signal of danger (Purdy). It not only indicates kidney degeneration and lessened function, but it causes a loss of the vital part of the blood. The other albumins which occur in the urine are called nucleo-albumins or proteids, such as the albumoses, peptone, mucin, catarrhal secretions, and excessive activity of the sexual and urinary glands. The nucleo-albumins are wayfarers along the urinary highways and bear a significance very different than does the presence of serum albumin, although albuminuria is often one of the manifestations of serious disease, not renal. Serum albumin in the urine, not due to hemorrhage into the urinary tract, indicates kidney lesion and as a concomitant result the non-elimination of the poisonous waste and decay of the system. The kidneys also eliminate poisons other than those elaborated by physiological processes. The kidneys are the great safety valves of our bodies, which are workshops of construction and repair and charnel houses of waste and decay from the cradle to the grave.

Albumose and peptone are formed during digestion of proteid foods, albuminous foods. There are different forms of albumoses, but there is no need of distinguishing them for our purpose in testing for albuminuria, since they behave much alike to chemical tests and have, so far as we yet know, an obscure pathological significance. The complete qualitative and quantitative chemical analysis of all of these substances have never yet been made by human hand. We will classify the albumoses and peptone as nucleo-albumins and learn to distinguish them from serum albumin. Serum albuminuria points to renal lesion, nucleo-albuminuria points to other lesions beyond or below the kidneys. The nucleo-albumins are found in the urine in tuberculosis, in acute rheumatism, in ulceration, endocarditis, in convulsions, also as a result of the injection of the organic antitoxins for the relief of certain diseases, due to microbic infection. Their presence warn us that severe tissue changes are in progress. Albuminuria in any form—nucleo- or serum—is a danger signal. Serum albumin and neucleo-albumin enjoy a blood relationship and frequently travel together in the urinary avenues. The kidneys not only eliminate the products of physiological waste and pathological débris, but also deleterious foreign matters which gain access to the system in various ways. The normal functionation of the kidneys is essential to health, and attencion to their action is demanded in all cases of sickness, whether it be due to kidney disease or any other malady.

Careful testing of the urine often supplies the missing link in the diagnostic chain. Testing for albumin in the urine is a chemical process, and chemistry is an exact science, but remember, in order to obtain exact results, your method must be exact, your chemicals pure and your manipulations faultless.

Purdy alleges that if the amount of serum albumin exceeds one-third of 1 per cent. it indicates a pathological condition. Jaksch, in his work on clinical diagnosis, says: "Serum albumin in notable quantity is never found in healthy urine." Serum albumin found in renal albuminuria is usually the result of inflammatory and degenerative changes in the kidney.

Samuel West, in a paper read before the London Medical Association, said: "That granular kidney—a form of Bright's disease—is a disease of more importance and exists more frequently than is generally supposed, since it is frequently unrecognised. It is often unsuspected until demonstrated by post mortem examination. It is found after death in other diseases, which might have resulted fatally, but for the presence of granular kidney. It is often overlooked; it is often unexpectedly found; it often supplies the missing link in obscure cases." Post mortem revealed granular kidney in a large percentage of 79 cases—excluding children under five years of age—brought to St. Bartholomew's Hospital, either dead or dying. 48 per cent. had chronic interstitial nephritis, which was the only cause of death in 16.8 per cent. It was the sole or contributing cause of death in 38.4 per cent. of these cases. 10 per cent. of 100 fatal cases of acute pneumonia showed, upon post mortem, granular kidney.

Dr. Purdy, author of practical urinalysis and urinary diagnosis, reports numerous cases of urinary disease, for which the sufferers sought relief in vain through two continents, to learn at last that they were dying of Bright's disease.

These things show the impoitance of urinary examination and the detection of albumin, hence a review of the more common tests employed is practically useful. All the common tests depend upon the formation of a cloud or precipitate, but the urine must be clear or the precipitate is obscured. The urine if clouded or opaque must be made clear by one or two filtrations through filter paper. Filtration does not clear the urine if the opacity or cloudiness be due to microbes or the result of decomposition. In this event, it has been advised to clarify the urine by the addition of chalk, magnesia, or a 5 per cent. solution of naphthalin, but as these substances derange the reactions of the chemical agents, it is better to use the Chambord-Pasteur filter. However, you will not be called upon to apply this process or to do more than use the paper filter, and even this is not often necessary.

To be exact, the sample for testing should be taken from the mixed product of twenty-four hours. While normal urine in the bladder is said to be both aseptic and antiseptic, yet it is very prone to infection and decomposition which renders it baneful. The total production for twenty-four hours should be accumulated in a glass vessel—the crystal water bottle fulfils the indication—with a capacity of at least three pints a few grains of chloral put into the bottle is a good preservative. A ready method of estimating the amount of solids is as follows: Multiply the last two figures of the specific gravity by the number of ounces passed in twenty-four hours and this product by 1.10.

Urinary insufficiency is indicated not by the bulk of water, but by the amounts of solids present in the secretion. There are two methods and many reagents used in testing urine for albumin. One method is by a mixture or diffusion of urine with the reagent. This is the method usually employed and is satisfactory unless there is a scant quantity of albumin. In this method the reagent is added directly by dropping it into the urine to the required amount. The other method is by contact. In the contact method the reagent is first placed in the test-tube and the urine is trickled along the tilted tube and floated over the surface of the reagent with contact, but not mixture. With this plan it is difficult to avoid blending or mixing of the fluid. It is not an easy matter to get contact without mixture; the commingling of the fluid will often occur in spite of careful manipulation.

In order to obviate this difficulty, the use of an ox-bow shaped test-tube, with a long and short arm, has been proposed. The curved portion is drawn into a capillary tube, about 1-16 of an inch in caliber. This formation lessens the tendency to admixture. Cover the mouth of the long arm with the finger and drop the urine into the short arm. Before uncovering the long arm, place the finger of the other hand over the mouth of the short tube and drop sufficient amount of reagent into the long arm to raise its level higher than that of the urine, then rotate the finger covering the short arm, so that the reagent shall pass through the space and force the urine to a level with itself. This manipulation brings the point of contact up into the enlarged portion of the tube and avoids blending of the fluids.

There is usually no difficulty presented in testing for albumin in the urine containing a large percentage of this substance, but when there are only traces which are obscured by other precipitates, great care is necessary and it is also essential to examine by several tests to be certain of the results. Urine is a variable and complex fluid and chemical reagents produce precipitates with other ingredients than albumin. It is said that reactions occur by many of the more delicate tests with normal urines from persons in sound health. This is stated to be due to mucus. It has been demonstrated, says Cammidge, that all urines contain small quantities of proteid material (nucleo-albumin) derived (supposedly) from the cells lining the urinary tract. Mörner demonstrated that urinary mucus was not identical with true mucus; that it is modified by combination with chondro-sulphonic and nuclinic acids; at all events it is classified as a nucleo-albumin or proteid, and so far as testing is concerned it need only be distinguished from serum albumin.

Under some circumstances and in inflammatory conditions of the urinary tract the substance may be present in sufficient quantity to respond to tests for renal albumin and obscure the results. Nucleo-albumin, unlike sero-albumin will precipitate with vegetable acids and the lighter or thinner the urine the greater the precipitate. Nucleo-like sero-albumin is precipitated by dilute mineral acids and unlike sero-albumin by alkalies, at least to a less degree. True mucus exists in small quantities in the urine, and its reactions are the same as those of the nucleo-albumins. In fact it is a nucleo-albumin. In making tests there is no purpose in distinguishing between mucus and nucleo-albumin, or differentiating serum albumin and globuline, for we do not know the pathological significance attached to one more than the other of these two substances (Cammidge). It is sufficient to know that they are serum albumin. The nebula or mucus cloud deposited by standing urine may be distorted epithelial cells or nucleo-albumin of another form.

The albumoses and peptone are found in the urine during suppurative processes and acute infectious diseases. They are the result of digestion of the albuminous foods and pass to the kidneys by way of the absorbents, lymphatics and the circulation of the blood. They are nucleo-albumins and precipitate with heat and mineral acids, obscure the serum albumins, but they disappear with boiling and reappear upon cooling. To eliminate the albumose, boil and filter while hot. Kuhn states that true peptone does not occur in the urine. Peptonuria is probably albumosuria and not an uncommon source of error when testing for renal or serum albumin. Various remedies produce conditions of the urine which obscure the tests for albumin by precipitation, such as the pine acids, the iodides, some of the balsams, while other drugs incite the elimination of serum-albumin itself. All these circumstances must be taken into consideration, several different tests applied and repeated if necessary to clear up all doubts in obscure cases.

Joles, in the *Baltimore Underwriter*, says that a reliable and available albumin test must be colorless; the reaction must show the minutest traces, so that its negative results indicate conclusively the absence of pathological traces of albumin, and finally the reagent must be independent of the compensation of the urine. He gives this formula:

| | | |
|---|---|---|
| ℞ | Hydr. chlo corros. | 10 |
| | Acid succinic | 20 |
| | Natr. chlor. | 10 |
| | Aqua dist. | 500 |

But it fails in alkaline urine, in the presence of mucin, phosphates and ammoniacal compounds and in the presence of iodides. All these substances must be disposed of before adding the reagent. To carry through this test requires the services of a chemical engineer and the paraphernalia of a laboratory. It is very delicate and will show albumin when present in 1 part to 120.000 of urine. It is too complicated for a bedside test.

Now it is in order to review ten or twelve of the many tests proposed:

1. *Heat.*—The heat test is generally employed. The reagent is obtainable in any household. It is delicate and under favoring conditions a very reliable test. One of the conditions for a favorable test is acid urine. In alkaline urine the albumin precipitate is obscured by other precipitates. You may acidulate the urine before heating or clear the field by acid after applying the heat. Heat precipitates the phosphates, if the urine be neutral or alkaline. Excess of acid interferes with the success of the test, since serum albumin is redissolved by excess of acid. You may get a precipitate of carbonates by heat, but acid will dissolve them, producing effervescence. Turbidity of cold urine is due to urates, nucleo-albumin or putrefactive germs. Heat will clear the turbidity due to urates, but not that due to organisms; chemicals may be employed; calcined magnesia or a few drops of caustic soda; after adding the soda shake and let it stand for a while and filter again. Slight turbidity, due to albumin plus heat is best seen by daylight; it may be overlooked by artificial light. Heat alone is not satisfactory as a test for albumin, but heat and acetic or nitric acid is the test usually employed.

2. *Heat and Acetic Acid.*—First take the reaction, if alkaline renders faintly acid. Boil the urine in a test-tube, and if no precipitate be formed add a little more acid; if no precipitate appears boil again. Heat alone will not precipitate all nucleo-albumins or proteids, but acetic acid will do so, especially in dilute urines and the turbidity produced thereby is indistinguishable in appearance from that due to small amount of serum albumin.

3. *Heat and Nitric Acid.*—Nitric acid does not so readily precipitate nucleo-albumin, but it forms with serum-albumin a coagulum, which is soluble with excess of acid. Bring the urine to the boiling point and add nitric acid drop by drop and very small amounts of albumin may be shown.

In concentrated urines nitric acid may produce powdery precipitate of urates which should be filtered off, or first dilute the urine and no urate precipitate will form. Precipitates are formed in the urine with nitric acid after the use of turpentine, benzoin, cubebs copaiba, storax, santal and other balsams, all of which are soluble in alcohol. Nitric acid will produce a precipitate in strongly bilious urine, also soluble with alcohol. These tests are delicate and simple, but there are many objections to them.

4. *Cold Nitric Acid* (Haller's test).—This is not as delicate as the others, but is sufficiently so for practical purposes. This test is applied by the contact method; a characteristic ring forms at the point of contact if serum-albumin be present. A second ring, less intense, forms a little higher up characteristic of nucleo-albumin, if that substance be present (Cammidge).

Instead of the second ring a diffuse haze sometimes forms in the presence of nucleo-albumin. Dilution of the urine diminishes the intensity of the serum albumin zone or ring and intensifies the nucleo-albumin opacity, which forms the second ring. The urates and resin acids are also precipitated by this test, but they are soluble with alcohol. Bilious urine forms colored rings with cold nitric acid contact. Peptone and the vegetable alkaloids are unaffected by the cold nitric acid test.

5. *Potassium-ferro-cyanide and Acetic Acid Test.*—This test is simple, delicate and extensively used. It is best used as a contact test. The mixture is one part of acetic acid and three parts of potassium ferrocyanide. At the point of contact with the urine, if serum-albumin be present a white ring quickly forms. Nucleo-albumin is not precipitated in the presence of an excess of potassium ferrocyanide. Concentrated urines should be diluted with distilled water. If the zone does not appear promptly wait a few minutes, as the precipitate forms slowly in the presence of a scant amount of albumin. This compound is not very stable and should not be heated. The phosphates, urates, alkaloids and peptones are not precipitated by this reagent, while the resin or pine acid are precipitated.

6. *The Salt and Acetic Acid Test.*—The salt solution is first added to the urine in excess; that is, the amount should be about half the volume of the urine and well shaken, then add the acetic acid and heat. The nucleo-albumin is not precipitated. This is a decided advantage. These reagents are found in every household and the test made by boiling over a lamp or stove. The fluid is then poured nto a glass, so as to be inspected by transmitted light. After diffusing the albumoses by heat, if the serum albumin precipitate be scanty, it is best found by means of daylight. The zone tests are preferable, but are not always available.

7. *Salicyl-sulphonic Acid Test.*—Cammidge claims that this is the freest from objections and the most convenient of all known tests; that it is cheap, clean and certain in its results. It may be used as a solid or in solution. It can be carried in a small tightly stoppered bottle (it is hygroscopic) and a crystal dropped into the urine to be tested. It acts equally well in acid and alkaline urines. Its use requires no special apparatus, except in the presence of albumose, when heat is necessary. Albumin and nucleo-albumins are quickly precipitated if present, but the nucleo-albumins are dissipated by heat, leaving the serum albumin diffused through the fluid, subject to inspection. It does not precipitate phosphates, urates, uric acid, bile, alkaloids or drugs. Its very delicacy is an objection since it renders albumin in quantities too small to be of pathological importance. The most serious objection, says Cammidge, is that it so readily precipitates nucleo-albumin or proteids which can not be distinguished from serum-albumin, except in the presence of heat. This objection is overcome by using Haller's cold nitric acid test, in doubtful cases. Again the inferior delicacy of the nitric acid test is a safeguard so that too much importance is not given to a mere trace of albumin. The nitric acid test alone may lead to error as a confirmatory test, but the errors are easily avoided. The salicyl-sulphonic acid sometimes gives a faint haze with normal urine, due to the presence of some form of nucleo-albumin, but with this exception all precipitates are due to albumin and albumoses.

8. *Trichloracetic Acid Test* precipitates everything except the albumoses.

9. *Picric Acid Test*, delicate, but uncertain.

10. Millard's reagent is composed of phenol, acetic acid and caustic potash.

11. Taneret's is composed of potassium, mercuric iodide and acetic acid.

12. Speigler's reagent, is composed of corrosive sublimate, alcohol and glucose.

There are many other tests.

I will resume this subject at some future meeting and demonstrate the use of the following tests:

1. Heat. Advantages and disadvantages.
2. Heat and nitric acid. Advantages and disadvantages.
3. Cold nitric acid. Advantages and disadvantages.
4. Potassium ferrocyanide and acetic acid. Advantages and disadvantages.
5. Salicyl-sulphonic acid. Advantages and disadvantages.

What are confirmatory tests?

Under what conditions are they indicated?

Serum albumin in the urine; its best test and significance.

Nucleo-albumin; its test and significance.
How would you distinguish between these two substances ?
What is the source of serum albumin found in albuminuria ?
What is the source of nucleo-albumin found in albuminuria ?

